# Biomolecular Characterization of Diazotrophs Isolated from the Tropical Soil in Malaysia

**DOI:** 10.3390/ijms140917812

**Published:** 2013-08-30

**Authors:** Umme Aminun Naher, Radziah Othman, Mohammad Abdul Latif, Qurban Ali Panhwar, Puteri Aminatulhawa Megat Amaddin, Zulkifli H Shamsuddin

**Affiliations:** 1 Institute of Tropical Agriculture, Universiti Putra Malaysia, 43400 Serdang, Selangor, Malaysia; E-Mails: naher39@gmail.com (U.A.N.); zulsham@upm.edu.my (Z.H.S.); 2 Bangladesh Rice Research Institute, Gazipur 1701, Bangladesh; E-Mail: latifbrri@gmail.com; 3 Department of Land Management, Universiti Putra Malaysia, 43400 Serdang, Selangor, Malaysia; E-Mails: drqurbanalip@gmail.com (Q.A.P.); puteriahawa@gmail.com (P.A.M.A.); 4 Department of Crop Science, Universiti Putra Malaysia, 43400 Serdang, Selangor, Malaysia

**Keywords:** diazotrophs, nitrogenase enzyme activity, indoleacetic acid, scanning electron microscopy, transmission electron microscopy, 16S rRNA

## Abstract

This study was conducted to evaluate selected biomolecular characteristics of rice root-associated diazotrophs isolated from the Tanjong Karang rice irrigation project area of Malaysia. Soil and rice plant samples were collected from seven soil series belonging to order Inceptisol (USDA soil taxonomy). A total of 38 diazotrophs were isolated using a nitrogen-free medium. The biochemical properties of the isolated bacteria, such as nitrogenase activity, indoleacetic acid (IAA) production and sugar utilization, were measured. According to a cluster analysis of Jaccard’s similarity coefficients, the genetic similarities among the isolated diazotrophs ranged from 10% to 100%. A dendogram constructed using the unweighted pair-group method with arithmetic mean (UPGMA) showed that the isolated diazotrophs clustered into 12 groups. The genomic DNA rep-PCR data were subjected to a principal component analysis, and the first four principal components (PC) accounted for 52.46% of the total variation among the 38 diazotrophs. The 10 diazotrophs that tested highly positive in the acetylene reduction assay (ARA) were identified as *Bacillus* spp. (9 diazotrophs) and *Burkholderia* sp. (Sb16) using the partial 16S rRNA gene sequence analysis. In the analysis of the biochemical characteristics, three principal components were accounted for approximately 85% of the total variation among the identified diazotrophs. The examination of root colonization using scanning electron microscopy (SEM) and transmission electron microscopy (TEM) proved that two of the isolated diazotrophs (Sb16 and Sb26) were able to colonize the surface and interior of rice roots and fixed 22%–24% of the total tissue nitrogen from the atmosphere. In general, the tropical soils (Inceptisols) of the Tanjong Karang rice irrigation project area in Malaysia harbor a diverse group of diazotrophs that exhibit a large variation of biomolecular characteristics.

## 1. Introduction

Rice plants are generally associated with several diazotrophs, and the *Azospirillum*, *Azoarcus*, *Enterobacter*, *Herbaspirillum*, *Burkholderia* and *Corynebacteria* genera are commonly associated with the rice rhizosphere. The use of biologically fixed nitrogen reduces the requirement for *N* chemical fertilizer and considerably ameliorates the environmental problems of NO_2_ emission and NO_3_ leaching. In addition to nitrogen fixation, diazotrophs also enhance crop growth through other processes. Certain microbes alter root development by producing growth hormones, such as auxin, ethylene and cytokinins, as well as volatile compounds [1]. Their overall impact on root morphogenesis increases the root surface area and volume, which help the plant to increase nutrient uptake and improve its performance under challenging environmental conditions.

Biological nitrogen fixation is an energy-consuming process; 16 moles of ATP are required to fix one mole of atmospheric nitrogen. Diazotrophs utilize root-exuded carbon compounds for their nitrogen fixation activity [2], and these carbon substrate utilization patterns are the definitive biochemical characteristics of these microorganisms. Indeed, the sugar uptake exhibited by diazotrophs is controlled genetically, and it is important to evaluate the sugar utilization preferences of microbes in addition to their nitrogen fixation and indoleacetic acid production.

The strain diversity of diazotrophs depends on the soil environment [3]. The tropical soil of Malaysia (Tanjong Karang) generally has a low pH (5.5) and thus favors low pH-tolerant diazotrophs. As the nitrogen fixation by diazotrophs is controlled by the *nif*H gene, most previous studies have evaluated strain diversity through the molecular detection of the *nif*H gene in soil isolates. All of the culturable and non-culturable diazotrophs in a soil solution can be detected using the Denaturing Gradient Gel Electrophoresis (DGGE) method [3,4]. But a few diazotrophs have been subjected to biofertilizer preparation. In this study, we used a conventional method (nitrogen-free medium) to isolate only culturable diazotrophs for their further identification and use in biofertilizer preparations. The 16S rRNA is an excellent molecular marker due to its highly conserved function and ubiquitous distribution and to the highly conserved to highly variable nature of the sequence [5]. In our study, we employed a molecular phylogenetic approach based on 16S rRNA sequences to identify pure potential isolates.

Rice is the third most cultivable crop in Malaysia, and it was presumed that rice-growing soils would harbor a large group of α- and β-subclass Proteobacteria. However, the diazotrophic strain diversity in the tropical soils of Malaysia has not yet been studied in depth. Therefore, the present study was conducted to identify potential efficient indigenous diazotrophs using molecular approaches and to evaluate their diversity, colonization efficiency and nitrogen fixation.

## 2. Results

### 2.1. Biochemical Characteristics of Diazotrophs

The bacterial strains isolated in the nitrogen-free semi-solid medium were evaluated for nitrogenase enzyme activity using an acetylene reduction assay (ARA). The ARA values ranged from 1.36 × 10^−6^ to 7.0 × 10^−11^ μmol C_2_H_4_ cfu^−1^·h^−1^, with the highest value found for isolate Sb35 (Table 1). Several other isolates, such as Sb6, Sb16, Sb17, Sb21, Sb23, Sb27 and Sb34 also exhibited high ARA values. The isolates with positive ARA values were considered nitrogen-fixing bacteria because nitrogen fixation is related to nitrogenase activity.

The potential for indoleacetic acid production by the diazotrophs was determined. In the presence of tryptophan, the isolated diazotrophs were able to produce high amounts of IAA, which varied from 15 mg·L^−1^ to 66.7 mg·L^−1^. The highest and lowest amounts of IAA were produced by strain Sb2, and Sb26 respectively (Table 1).

In this study, Jensen-CMC plates were assayed to determine the cellulase activity. Plates were spotted with aliquots of diazotroph broth, and the presence of a clear halo zone after staining with 0.1% Congo red indicated cellulase activity. Twenty one of the isolates were positive for cellulase activity (Table 1). The nitrogen fixation is needed the energy source and diazotrophs consume carbon substrates from the rhizosphere of the plants. According to the higher values of ARA and IAA production, 10 isolates were evaluated for sugar utilization. The tested diazotrophs were able to utilize approximately 69.3%–100% of the supplied sugar (glucose, fructose, arabinose, sucrose and galactose).

### 2.2. Molecular Characteristics of Diazotrophs

#### 2.2.1. Cluster Analysis

**Figure 1:**
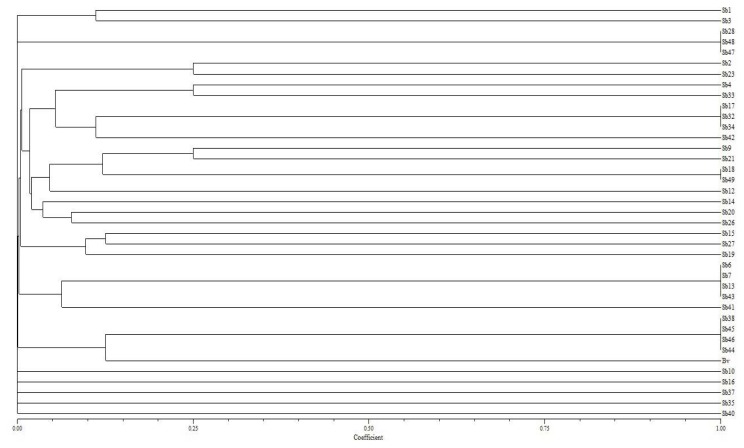
The dendogram of cluster analyses of isolated diazotroph based on REP-PCR marker [6]. Dendogram constructed by Jaccard similarity coefficient matrix by unweighted pair-group method with arithmetic mean (UPGMA) method.

The rice soil contains a diverse group of diazotrophs. Using Jaccard’s genetic similarity coefficient, we established that different levels of genetic variation exist among the isolates, ranging from 0.010 to 1.0 (Table 2). The UPGMA cluster analysis of the Jaccard’s similarity coefficient was used to generate a dendogram (Figure 1), illustrating the overall genetic relationship among the diazotrophs. According to this analysis, 38 diazotrophs were grouped into twelve major clusters at a coefficient level of 0.001. Each group of isolates consisted of identical bands. Cluster I contained two diazotrophs (Sb1 and Sb3) and Cluster II contained three diazotrophs, Sb28, Sb48 and Sb49. Cluster III had only two isolates, Sb2 and Sb23. The largest number of diazotrophs was in cluster IV, including Sb4, Sb9, Sb12, SB14, Sb17, Sb18, Sb20, Sb21, Sb26, Sb32, Sb33, Sb34, Sb42, and Sb49. Cluster V existed three (Sb15, Sb19 and Sb27) on the other hand Cluster VI contained five diazotrophs (Sb6, Sb7, Sb13, Sb41 and Sb43). Whereas cluster VII contained four diazotrophs and remaining clusters VIII to XII contained one isolate each. A large variation was observed among the diazotrophs, which could be due to the different soil types within the study area (seven soil types or series).

#### 2.2.2. Principal Component Analysis (PCA)

The first four principal components (PC) accounted for 52.28% of the total variation among the 38 genotypes of diazotrophs. PC1, PC2, PC3 and PC4 accounted for 5.04%, 10.27%, 16.62% and 20.35% of the variation, respectively (Table 3).

**Figure 2:**
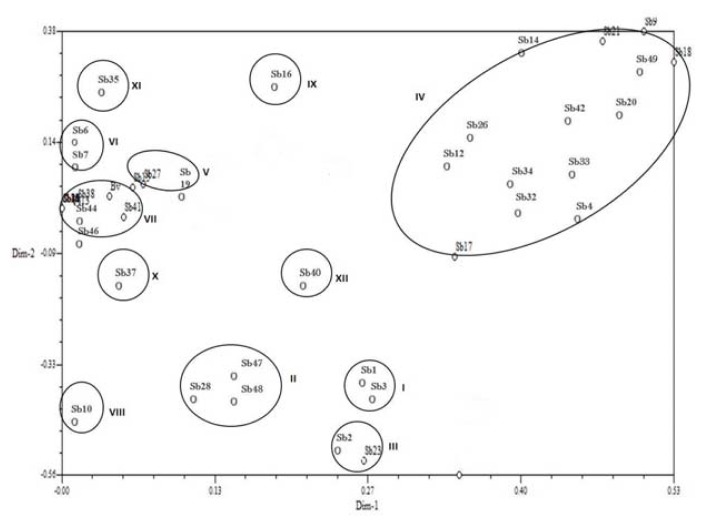
Principal component analysis (PCA) generated from genomic DNA rep-PCR which showed genetic similarity distance of 38 isolates.

In principal component analysis, 38 isolates also formed 12 clusters in a two dimensional graph (Figure 2). Here most of isolates followed the same clustering groups as shown in cluster analysis with little exception in clusters i.e. VI and VII. In cluster analysis group VI contained 5 isolates (Sb6, Sb7, Sb13, Sb41 and Sb43) while in PCA group it contained only two isolates (Sb6 and Sb7). The analysis supplemented the cluster analysis. The diazotrophs genotypes, Sb18 (0.53), Sb9 (0.51), Sb21 (0.47), Sb17 and Sb4 (0.34), Sb19 and Sb14 (0.18), Sb12 (0.17), Sb12 (0.13), Sb20 (0.08), Sb15 (0.06) and Sb13 (0.01), were positively associated with PC1 and showed high similarity values (Table 3).

**Figure 3:**
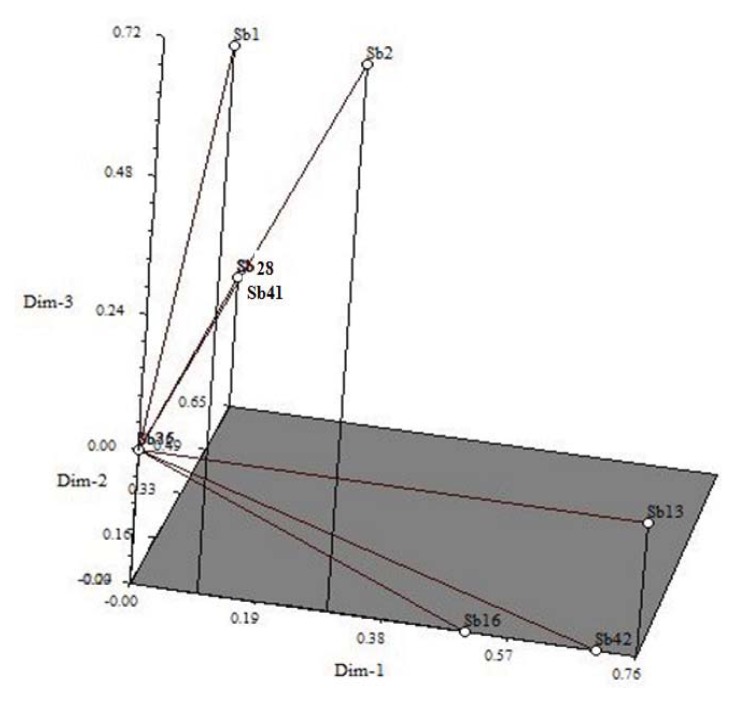
Principal component analysis for biochemical characteristics of 10 selected isolates. Sb1 accession JQ820251, Sb2 accession JQ820252, Sb6 accession JQ820253, Sb13 accession JQ820254, Sb16 accession JQ820255, Sb26 accession JQ820256, Sb28 accession JQ820257, Sb35 accession JQ820258, Sb41 accession JQ820259, and Sb42 accession JQ82026.

Seven biochemical properties of the ten selected isolates were subjected to PCA to determine the genetic relatedness among these isolates (Figure 3); approximately 85% of the total genetic variation was present among these 10 strains (Table 4). The first three components accounted for 51%, 22%, and 12% of the variation. Strains Sb1, Sb2, Sb16, Sb13 and Sb42 exhibited distance from the centroid, whereas the others (Sb6, Sb26, Sb28, Sb35 and Sb41) were close to the centroid (Figure 3). Among these strains, Sb1 and Sb2 utilized 100 percent of the supplied glucose. A preference for galactose utilization (100%) was found for Sb13, Sb16 and Sb42. In contrast, Sb6, Sb28 and Sb41, which were placed around the centroid, demonstrated a preference for fructose over other sugars. The first principal component accounted for 51% of the variation, with glucose (0.052) and fructose (0.030) positively contributing. Arabinose (0.033) and galactose (0.55) contributed positively to the second principal component, whereas IAA (0.25), glucose (0.16) and fructose (0.26) contributed positively to the third principal component.

#### 2.2.3. Identification of Diazotrophs

**Figure 4:**
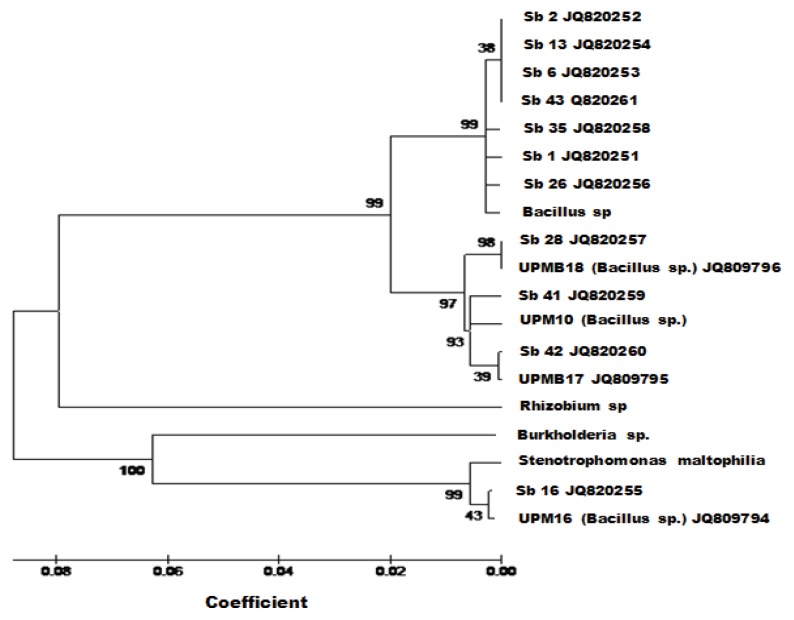
Phylogenetic tree with bootstrap values. Tree constructed using Neighbor-Joining (NJ) method. Sb1, Sb2, Sb6, Sb13, Sb16, Sb26, Sb28, Sb35, Sb41, Sb42, UPMB10 (*Bacillus* sp.), UPMB16 (*Bacillus* sp.), UPMB17 (*Bacillus* sp.), UPMB18 (*Bacillus* sp.), *Rhizobium* sp. and *Stenotrophomonas maltophilia.*

In this study, we used partial gene sequences from β-subclass Proteobacteria (*Rhizobium*, *Bacillus* and *Burkholderia* genera and *Stenotrophomonas maltophilia*) as references to determine the phylogenetic relationships among the tested isolates. Because some of the physiological characteristics of the isolated bacteria were identical (data not shown) to those of this strain, we selected *Stenotrophomonas maltophilia* as the reference strain. The Neighbor-Joining tree was subjected to the numerical re-sampling by bootstrapping, and the resulting bootstrap values are shown at the tree branch nodes. Each value represents the number of times (out of 1000 replicates) that the represented groupings occurred in the resamplings. The consensus tree showed 99% confidence levels between nine diazotrophs (Sb2, Sb13, Sb6, Sb43, Sb1, Sb26, Sb28, Sb41 and Sb42) and *Bacillus* sp. (β-subclass of Proteobacteria*)*, whereas Sb16 and *Burkholderia* sp. had a 100% confidence level (Figure 4). Our results indicate that the tropical soils (above-stated seven soil series) of Malaysia are dominated by the β-subclass of Proteobacteria.

#### 2.2.4. Colonization and Efficiency of Biological Nitrogen Fixation (BNF) by Sb16 and Sb26

**Figure 5:**
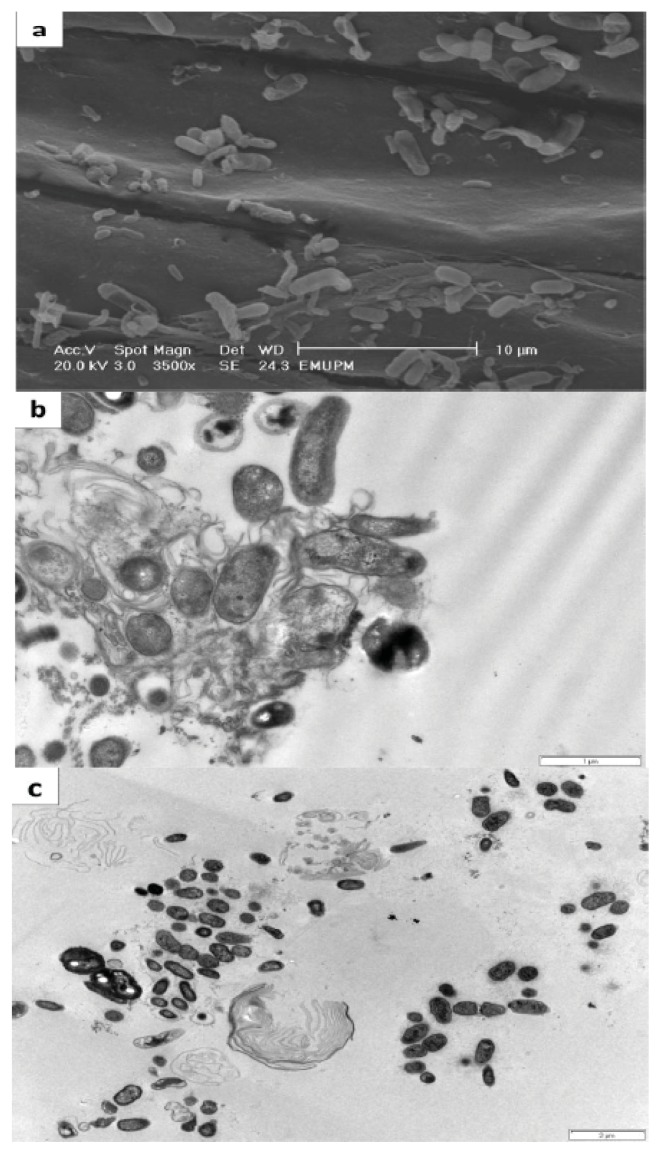
Scanning electron microscopy (SEM) and transmission electron microscopy (TEM) micrograph of inoculated rice genotypes. (**a**) SEM micrograph, surface colonization of Sb42 inoculated with MR219 rice; (**b**) TEM micrograph, Sb26 inside the root cell of Mayang Segumpal rice; (**c**) TEM micrograph, Sb16 inside the root cell of MR219 rice.

Based on our results, Sb16 was identified as *Burkholderia* sp. and Sb26 was identified as *Bacillus* sp. Under *in vitro* conditions, both of the strains exhibited a high nitrogenase activity and produced a substantial amount of IAA. Based on these characteristics, these two strains were selected for root colonization and BNF studies. Scanning and transmission electron microscopy proved that both of the diazotrophs were able to colonize the root surface and interior (Figure 5). Both diazotrophs also have the potential to fix atmospheric nitrogen and contributed approximately 22%–24% of the total nitrogen in the plant tissue. According to a ^15^N isotope study, diazotrophs can fix approximately 10 kg·N·ha^−1^–12 kg·N·ha^−1^ within a 60 day period (Table 5). The results of some ^15^N studies showed that the proportion of nitrogen derived from the atmosphere and the amount of N_2_ fixed in plant tissues varies across species.

## 3. Discussion

Several diazotrophs were isolated from tropical soils of Malaysia. The isolates were capable for the nitrogenase enzyme activity proved by the acetylene reduction assay (ARA). The isolates with positive ARA values were considered nitrogen-fixing bacteria because nitrogen fixation is related to nitrogenase activity [7]. The promotion of plant growth by diazotrophs is an effect of nitrogen fixation and growth hormone production [8]. Therefore, the potential for indoleacetic acid production by the diazotrophs was evaluated. Diazotrophs are free-living nitrogen-fixing bacteria, and some are endophytic. Diazotrophs that produce cellulase can degrade cell walls, which is one potential mechanism for entry into root tissue [9]. Because nitrogen fixation is an energy-consuming process, diazotrophs utilize rhizosphere carbon substrates as an energy source to support nitrogen fixation, and approximately 64%–86% of the carbon released by the plant into the rhizosphere is consumed by microorganisms [10].

Rice root exudates generally include simple sugars, such as glucose, fructose, mannose, xylose, arabinose, galactose and sucrose [11]. However, different isolates demonstrated different carbon source requirements, and physiologically distinct organisms were enriched on each carbon source tested [12]. Rice soil harbors a diverse group of diazotrophs. Ueda *et al.* [13] constructed a clonal library of PCR-amplified *nif*H sequences, which revealed the great diversity of uncharacterized diazotrophs in the rice rhizosphere. The strain diversity of free-living diazotrophs depends on several soil environmental factors, such as the soil pH, C abundance and N availability [3]. Isolates formed various clusters with the various biochemical properties. The diazotrophs were found to differ in the total genetic variation through the principal component accounted and varied for the consumption of sugar as a carbon source. Most of the diazotrophs followed the same clustering groups like cluster analysis with little exception in clusters. Therefore, PCA almost supplemented the cluster analysis. To confirm the accuracy of the grouping of isolates or germplasms several researchers use more than one multivariate analysis [14–16]. A previous study also showed that several diazotrophs have a preference for a particular sugar as a carbon source [17]. Sugar preference is controlled by the ChvE protein, a periplasmic sugar-binding protein that is homologous to the *Escherichia coli*galactose-binding protein. Diazotrophs containing a ChvE homologous protein showed strong preferences for d-galactose, l-arabinose, and d-fructose [17].

Ribosomal ribonucleic acids are excellent markers for the clarification of bacterial phylogeny [5]. After the partial gene sequences *Rhizobium*, *Bacillus* and *Burkholderia* genera and *Stenotrophomonas maltophilia* were identified. In Korea, *Stenotrophomonas maltophilia* was isolated from rice soil and identified as a nitrogen-fixing bacterium [18]. The recognition and known diversity of diazotrophic β-proteobacteria have increased significantly in the last decade [19]. The first diazotrophic species of the *Burkholderia* genus identified was *B. vietnamiensis*, which was isolated from the rice rhizosphere in a screen for nitrogen-fixing bacteria [20]. A contribution to biological nitrogen fixation and association with rice plants have also been documented for *Bacillus* spp.

Some of the isolates based on their characteristics, proved that the diazotrophs were able to colonize the root surface and interior. Moreover, diazotrophs have the potential to fix atmospheric nitrogen (22%–24%) by the ^15^N studies. It was found that free-living diazotrophs can fix between 0 kg·ha^−1^ year ^−1^ and 60 kg·ha^−1^year^−1^ of atmospheric nitrogen [21]. According to Mirza *et al.* [22] and Malik *et al.* [23] approximately 59% of the N_2_ in rice ecosystems is derived from the atmosphere. In another study, Govindarajan *et al.* [24] reported that 40.4% of the N_2_ in a rice ecosystem was fixed by *Burkholderia vietnamiensis* under glasshouse conditions. However, the nitrogen-fixing potential strongly depends on the type of diazotroph and the nature of its association with the plant.

## 4. Experimental Section

### 4.1. Collection and Isolation of Diazotrophs

Plant and soil samples were collected from seven soil types or series (Jawa, Sedu, Bakau, Bernam, Serong, Organic Clay & Muck and Brown Clay) from the Tanjong Karang rice irrigation project area. All of the soils are considered Inceptisols (USDA soil taxonomy). The diazotrophs were from the rhizosphere and non-rhizosphere soil were isolated using a nitrogen-free (Nfb) semi-solid malate medium [25] per litre consisting of 5 g malic acid, 0.5 g K_2_HPO_4_, 0.2 g MgSO_4_·7 H_2_O, 0.1 g NaCl, 0.02 g CaCl_2_, 0.5% bromothymol blue in 0.2 N KOH (2 mL), 1.64% Fe-EDTA solution (4 mL) and 2 g agar. A total of 38 diazotrophs were isolated. Selected soil chemical properties are given in Table 6.

### 4.2. Estimation of Nitrogenase Enzyme Activity

The nitrogenase activity was assayed using the acetylene reduction assay (ARA). A 1 mL aliquot of the diazotroph culture was transferred to an airtight 30 mL bottle containing 10 ml of *N*-free semi-solid malate (Nfb) medium. After pellicle formation, the bottles were injected with 5% *v*/*v* acetylene gas, and the same volume of air was removed simultaneously. The bottles were incubated at 30 °C for 24 h [26], and 1.0 mL of gas was withdrawn and transferred to a vacuum tube (Vacutainer™ 7 mL). The presence of ethylene was assayed using a G-300 gas chromatograph (GC) equipped with FID detector. The rate of N_2_ fixation was expressed as the quantity of ethylene accumulated (μmol C_2_H_4_ cfu^−1^ h^−1^) based on the standard curve and peak area percentage.

### 4.3. Determination of Indoleacetic Acid (IAA) Production

The isolates were inoculated in Jensen’s broth (Sucrose 20 g, K_2_HPO_4_ 1 g·L^−1^, MgSO_4_ 7H_2_O 0.5 g·L^−1^, NaCl 0.5 g·L^−1^, FeSO_4_ 0.1 g·L^−1^, NaMoO_4_ 0.005 g·L^−1^, CaCO_3_ 2 g·L^−1^) containing 2 mg·mL^−1^ tryptophan and incubated at 29 ± 1 °C for 72 h. Approximately 2 mL of the culture solution was centrifuged at 7000 rpm for 7 min, and the supernatants were used to determine the IAA concentration. A 1 mL aliquot of the supernatant was mixed with 2 mL of Salkowski’s reagent as described by Gordon and Weber [27], and the absorbance was measured using a spectrophotometer at 530 nm. The concentration of IAA was determined using a standard graph.

### 4.4. Determination of CelluSlase Activity

The cellulase activity was evaluated using Jensen’s agar plates with 0.1% carboxymethyl cellulose [28]. The plates were spot-inoculated with 10 μL of liquid culture. After 24 h of incubation, the colonies were streaked, washed with sterile water and discarded. The plates were stained with 0.1% Congo red solution for 30 min and then rinsed with 1 M NaCl. A positive cellulose-degrading enzyme reaction was indicated by a clear halo zone on the plate.

### 4.5. Determination of Sugar Consumption

The carbon- and nitrogen-free nutrient culture broth used was modified from Egener *et al.* [29] using 15 g of different sugars (glucose, fructose, sucrose, galactose and arabinose). The composition of the broth per liter was: KH_2_PO_4_, 1.5 g; K_2_HPO_4_, 0.33 g; K_2_SO_4_, 0.2 g; ferric citrate, 13 mg; CaCl_2_·2H_2_O, 0.4 g; MgCl_2_, 0.4 g; Na_2_MoO_4_·2H_2_O, 2 mg; H_3_BO_3_, 3 mg: MnSO_4_·H_2_O, 2 mg; ZnSO_4_·7H_2_O, 0.2 mg and CuSO_4_·5H_2_O, 0.1 mg. After 36 h of incubation, the cultures were filtered through 0.2 μM pore syringe filters, and 20 μL of each sample was separated using high-performance liquid chromatography (HPLC, 1100 Series, Agilents, Harlow Scientific, Arlington, Middlesex County, MA, USA, 2002). The amount of residual glucose, fructose, sucrose and arabinose was determined using an Apex column at 60 °C and a refractive index (R.I.) detector. Acetonitrile (75%) was used as the mobile phase at a flow rate of 1.8 mL·min^−1^. The amount of sugar consumption was determined by subtraction from the initial substrates as follows:

(1) $$C=S_{t0}-S_{t}$$

where, *C* represents the amount of substrate consumed, *S_t0_* represents the substrate added at the initial time and *S_t_* represents the substrate remaining in the culture solution at each sampling time.

### 4.6. Strain Diversity and Diazotrophs Identification

#### 4.6.1. DNA Extraction and Primers

The bacterial genomic DNA was extracted from the pure bacterial culture using the GF^−1^ bacterial DNA extraction kit, Vivantis, Malaysia. Rep-PCR was performed using the primers published [30]: REP IR, 5′-IIIICgICgICATCIggC-3′, and REP 2I, 5′-ICgITTATCIggCCTAC-3′.

#### 4.6.2. PCR Protocols and Gel Electrophoresis

The strain diversity was evaluated by rep-PCR genomic fingerprinting. The 25 μL PCR reaction volume consisted of the following: 5 μL 5× Gitschier buffer, 2.5 μL DMSO, 1.25 μL dNTPs (1:1:1:1), 1 μL each primer, 1 μL Taq DNA polymerase and 1 μL of purified genomic DNA. The thermal cycler (MJ Mini personal Thermal Cycler, Bio-Rad, Model-PTC-1148) conditions were as follows: 95 °C for 6 min and 30 cycles of 94 °C for 1 min, 40 °C for 1 min, and 65 °C for 8 min, followed by 1 cycle at 65 °C for 16 min. The PCR reaction ended with an extension temperature of 65 °C for 8 min, and the products were stored at −20 °C until separation by 1% agarose electrophoresis in 10× TAE buffer.

#### 4.6.3. Strain Identification by Partial Gene Sequencing

Ten isolates with high ARA values were selected for partial 16S rRNA gene sequencing analysis. The DNA was extracted from pure isolates using the QIAamp Genomic DNA Mini Kit UK AS. The rep-PCR was performed using the forward primer *8F*, 5′-AGA GTT TGA TCC TGG CTC AG-3′ and the reverse primer *1492R*, 5′-GGT TAC CTT ACG ACT T-3′. The primers selected for this study amplify sequences from both symbiotic and free-living nitrogen-fixing bacteria. The PCR products were purified using the Gene JET RNA purification kit (Thermo Scientific™ Fermentas GeneJET RNA Purification Kit, Waltham, MA, USA) and sequenced by First BASE laboratories, Malaysia. The partial gene sequences were aligned using the ClustalW package [31]. An unrooted phylogeny tree was constructed using the Neighbor-Joining method [32]. Partial sequences for the strains (*Bacillus* sp., GenBank JN695718.1, *Burkholderia* sp. GenBank AF219125.1 and *Stenotrophomonas maltophilia*, GenBank HQ219979.1) were obtained as references. The topology of the distance tree was tested by re-sampling the data using 1000 bootstrap replicates [33]. The phylogenetic analyses were conducted in MEGA4 [34]. The sequences obtained were deposited in the European Molecular Biology Laboratory data bank (accession number JQ820251 to JQ820260).

#### 4.6.4. Determination of Nitrogen Fixation (^15^N Isotope Dilution Technique)

Two strains, Sb16 and Sb26, were selected for biological nitrogen fixation (BNF) assessment in a greenhouse study. The popular modern rice variety MR219 and the local accession Mayang Segumpal were inoculated with the selected diazotrophs, and the N_2_ fixation rate was estimated using the ^15^N isotope dilution method [35]. Plant tissue-nitrogen was determined by semi-micro Kjeldahl method [36]. Soil pH was measured in soil; water (1:2.5) extract using PHM210 Standard pH meter at 30 °C [37] and total soil carbon determined by infrared absorption method (LECO CR-412), The %^15^N (a.e.) in plant part was estimated using the following formula Warembough [38]:

(2) %N(a.e)in each plant part=%N abundance-0.3663 % N15(natural abundance)

assuming that the atmosphere contains 0.3663% ^15^N.

The proportion of N derived from the atmosphere had been calculated as (Warembough [38]):

(3) %Ndfa=[1-%N15 a.e.in the fixing system%N15a.e.in the non fixing system]×100

The total N_2_ fixation in plant tissue was calculated as (Warembough [38]):

(4) $$N_{2}fixed\;(mg)=\frac{\%\;Ndfa\times total\;N\;content}{100}$$

Rice seeds were surface sterilized followed by Amin *et al*. [39]. Rice seeds were agitated in 70% ethanol (5 s). The ethanol was discarded and the seeds washed in sodium hypochlorite solution comprising 3% Chlorox TM (2.6% NaOCl), with a few drops of Tween 20. The seeds were rinsed with sterile water followed by 2% sodium thiosulphate solution to neutralize chloramine residue. Seven days old seedlings (4 seedlings) were transplanted into each pot containing 10 kg of soil. Seedlings were inoculated with washed cell of diazotrophs (Sb16 *Burkholderia* sp. and Sb26 *Bacillus* sp. at 10^6^ cfu after 3 days of transplanting. Dead cells (By inoculum sterilization) were applied at control plant. Exactly 0.1 g of ^15^N urea with atomic excess (10.18) was diluted with distilled water and poured uniformly in each pot (3 days before planting to stabilize the soil). Blanket doses of P_2_O_5_, and K_2_O from sodium phosphate (monobasic), and potassium chloride at the rates of 60 and 40 kg·ha^−1^ equivalent, respectively, were applied to each pot.

#### 4.6.5. Observation of Root Colonization Using SEM and TEM

Root colonization was studied using scanning electron microscope (JSM-5610LV SEM, JEOL, Datum Ltd., Tokyo, Japan, 1996) and transmission electron microscopy (Leo 912AB EFTEM, ITEM software, Soft Imaging Systems, Omega, Zeiss, Oberkochen, Germany, 1999).

### 4.7. Statistical Analysis

The biochemical analyses and glasshouse experiments were arranged in completely randomized designs with 5 replicates. The quantitative results were subjected to an analysis of variance (ANOVA), and significance at the 5% level was tested by Tukey’s studentized range using SAS statistical program version 9.1 (SAS Institute, Cary, NC, USA, 2008). The genomic DNA was analyzed using NTSYS-pc software to assess the genetic relatedness of the 38 isolated diazotrophs. The coefficients of genetic similarity for all of the pair-wise comparisons were computed using the Jaccard’s coefficient [40], and the similarity matrix was subjected to a cluster analysis using the unweighted pair-group method with arithmetic mean (UPGMA) to produce a dendogram. PCA was also performed for the isolates and their biochemical properties. More than one multivariate technique is required to represent the results more clearly and it is obvious from the results of many researchers [41,42].

## 5. Conclusions

The tropical soils (rice soil) of Malaysia are dominated by β-subclass Proteobacteria: 9 diazotrophs were identified as *Bacillus* spp. and one was identified as a *Burkholderia* sp. (Sb16). Isolates Sb16 and Sb26 (*Bacillus* spp.) were able to colonize the rice rhizosphere and the interior of the root tissue. Scanning and transmission electron micrographs clearly showed that both of the diazotrophs were able to colonize the root surface and interior. These strains had the potential to fix atmospheric nitrogen and contributed approximately 22%–24% atmospheric nitrogen to the total plant tissue nitrogen. In addition, the ^15^N isotope analysis also proved that both diazotrophs can fix approximately 10 kg N ha^−1^–12 kg N ha^−1^ within a 60 days period. Four principal components were identified among the 38 isolated diazotrophs and were accounted for 52.46% of the total genetic variation, confirming that the evaluated soil harbors a diverse group of diazotrophs.

## Figures and Tables

**Table 1:** Biochemical properties of diazotrophs isolated from Tanjong Karang rice growing area.

Isolate	ARA (μmol·C_2_H_4_^−1^·cfu^−1^·h^−1^)	Cellulose degradation	IAA production (mg·L^−1^)	Sugar consumption (%)				

				Glucose	Fructose	Sucrose	Arabinose	Galactose
Sb1	1.9 × 10^−8^	−	26.0	100.0	98.25	72.00	89.00	85.00
Sb2	1.6 × 10^−9^	+	66.7	100.0	96.00	79.33	76.32	82.33
Sb3	2.3 × 10^−10^	−	48.0	nd	nd	nd	nd	nd
Sb4	nd	+	32.0	nd	nd	nd	nd	nd
Sb6	1.3 × 10^−7^	+	57.6	90.54	100.0	86.84	83.00	98.00
Sb7	nd	+	63.0	nd	nd	nd	nd	nd
Sb9	nd	+	34.02	nd	nd	nd	nd	nd
Sb10	2.7 × 10^−10^	−	34.2	nd	nd	nd	nd	nd
Sb12	1.23× 10^−7^	+	16.49	nd	nd	nd	nd	nd
Sb13	3.1 × 10^−9^	−	54.2	97.33	96.00	96.00	100.0	100.0
Sb14	2.3 × 10^−7^	−	32.03	nd	nd	nd	nd	nd
Sb15	1.8 × 10^−6^	−	24.41	nd	nd	nd	nd	nd
Sb16	1.4 × 10^−7^	+	59.5	90.67	76.00	80.00	98.67	100.0
Sb17	2.1 × 10^−7^	−	22.43	nd	nd	nd	nd	nd
Sb18	2.7 × 10^−10^	−	43.0	nd	nd	nd	nd	nd
Sb19	1.7 × 10^−7^	−	17.14	nd	nd	nd	nd	nd
Sb20	3.9 × 10^−9^	−	52.0	nd	nd	nd	nd	nd
Sb21	2.3 × 10^−7^	−	13.21	nd	nd	nd	nd	nd
Sb23	1.7 × 10^−7^	−	16.32	nd	nd	nd	nd	nd
Sb26	2.9 × 10^−10^	+	15.0	96.00	93.33	69.33	88.00	86.91
Sb27	1.8 × 10^−7^	−	nd	nd	nd	nd	nd	
Sb28	2.1 × 10^−9^	+	51.0	94.67	100.0	94.67	93.33	75.00
Sb32	5.4 × 10^−10^	+	62.0	nd	nd	nd	nd	nd
Sb33	1.8 × 10^−10^	+	22.0	nd	nd	nd	nd	nd
Sb34	4.9 × 10^−7^	+	47.0	nd	nd	nd	nd	nd
Sb35	1.36 × 10^−6^	−	30.4	89.00	96.76	79.32	90.00	84.54
Sb37	2.5 × 10^−10^	+	49.0	nd	nd	nd	nd	nd
Sb38	6.1 × 10^−11^	+	31.0	nd	nd	nd	nd	nd
Sb40	1.2 × 10^−8^	−	18.04	nd	nd	nd	nd	nd
Sb41	9.1 × 10^−10^	+	53.0	89.53	100.0	81.82	96.00	96.33
Sb42	6.1 × 10^−11^	+	60.0	91.00	98.00	96.00	85.00	100.0
Sb43	4.2 × 10^−12^	−	21.0	nd	nd	nd	nd	nd
Sb44	5.1 × 10^−11^	−	15.61	nd	nd	nd	nd	nd
Sb45	7.0 × 10^−11^	+	18.1	nd	nd	nd	nd	nd
Sb46	6.1 × 10^−10^	+	41.0	nd	nd	nd	nd	nd
Sb47	2.2 × 10^−9^	+	24.0	nd	nd	nd	nd	nd
Sb48	6.3 × 10^−11^	+	20.0	nd	nd	nd	nd	nd
Sb49	1.1 × 10^−11^	+	22.4	nd	nd	nd	nd	nd

nd, not done; +, positive; −, negative (cellulose degradation).

**Table 2:** Intra and inter similarity matrix among clusters of 38 diazotrophs using genomic DNA rep-PCR.

	Cluster I	Cluster II	Cluster III	Cluster IV	Cluster V	Cluster VI	Cluster VII	Cluster VIII	Cluster IX	Cluster X	Cluster XI	Cluster XII
Cluster I	0											
Cluster II	0.039	0										
Cluster III	0.021	0.024	0									
Cluster IV	0.018	0.026	0.022	0								
Cluster V	0.022	0.030	0.039	0.018	0							
Cluster VI	0.019	0.043	0.022	0.021	0.010	0						
Cluster VII	0.017	0.044	0.025	0.020	0.021	1.000	0					
Cluster VIII	0.019	0.012	0.026	0.021	0.011	1.000	1.000	0				
Cluster IX	0.018	0.043	0.025	0.025	0.010	1.000	1.000	1.000	0			
Cluster X	0.019	0.029	0.024	0.022	0.010	1.000	1.000	1.000	1.000	0		
Cluster XI	0.018	0.044	0.025	0.021	0.103	1.000	1.000	1.000	1.000	1.000	0	
Cluster XII	0.019	0.029	0.022	0.010	0.03	1.000	1.000	1.000	1.000	1.000	1.000	0

**Table 3:** Component loading of the first four principal components (PC) for 38 diazotrophs by genomic DNA rep-PCR analysis.

Strains	First four principle components			

	PC1	PC2	PC3	PC4
Variation accounted for	5.04	10.27	16.62	20.35
Sb1	0.00	0.00	0.00	0.00
Sb2	0.17	−0.38	−0.43	0.23
Sb3	0.00	0.00	0.00	0.00
Sb4	0.34	−0.56	0.04	−0.07
Sb6	0.01	0.00	0.02	−0.03
Sb7	0.02	0.00	0.03	−0.02
Sb9	0.51	0.38	−0.33	−0.15
Sb10	0.00	0.00	0.00	0.00
Sb12	0.13	0.08	0.01	0.01
Sb13	0.01	0.00	0.03	−0.02
Sb14	0.18	0.11	0.17	0.22
Sb15	0.06	0.05	0.14	0.52
Sb16	0.00	0.00	0.00	0.00
Sb17	0.34	−0.10	0.46	−0.19
Sb18	0.53	0.31	0.02	0.03
Sb19	0.18	0.12	0.17	0.56
Sb20	0.08	0.01	0.22	−0.06
Sb21	0.47	0.35	−0.33	−0.15
Sb23	0.26	−0.53	−0.42	0.21
Sb26	0.23	0.07	0.29	−0.01
Sb27	0.07	0.05	0.15	0.57
Sb28	0.00	0.00	0.00	0.00
Sb32	0.31	−0.11	0.44	−0.18
Sb33	0.35	−0.44	0.32	−0.20
Sb34	0.33	−0.12	0.45	−0.17
Sb35	0.00	0.00	0.00	0.00
Sb37	0.00	0.00	0.00	0.00
Sb38	0.01	0.01	0.04	0.06
Sb40	0.00	0.00	0.00	0.00
Sb41	0.05	−0.02	0.12	−0.06
Sb42	0.12	−0.03	0.30	−0.12
Sb43	0.01	0.00	0.03	−0.03
Sb44	0.01	0.01	0.03	0.05
Sb45	0.02	0.00	0.03	0.09
Sb46	0.03	0.01	0.02	0.08
Sb47	0.01	0.00	0.01	0.00
Sb48	0.00	0.01	0.01	0.00
Sb49	0.23	0.26	0.02	0.01
*Bv	0.04	0.03	0.07	0.11

* Bv, *Burkholderia* sp. (Gene bank AF219125.1).

**Table 4:** Component loading of the first three principal components (PC) for * 10 diazotrophs by genomic DNA rep-PCR analysis.

Variable	PC1	PC2	PC3
proportion	0.51	0.22	0.12
ARA	−0.039	−0.751	−0.276
IAA	−0.893	−0.009	0.255
Glucose	0.052	−0.077	0.167
Fructose	0.030	−0.244	0.268
Sucrose	−0.383	−0.253	−0.316
Arabinose	−0.007	0.033	−0.742
Galactose	−0.223	0.552	−0.329

* Sb1 accession JQ820251, Sb2 accession JQ820252, Sb6 accession JQ820253, Sb13 accession JQ820254, Sb16 accession JQ820255, Sb26 accession JQ820256, Sb28 accession JQ820257, Sb35 accession JQ820258, Sb41 accession JQ820259, Sb42 accession JQ820260.

**Table 5:** Effect of diazotroph inoculation on MR219 (HYV rice) and Mayang Segumpal (local rice accession).

Rice genotype	Diazotrophs	^15^N (%a.e.)	Tissue N (%)	N fixed (mg·plant^−1^)	% Nfda	N fixed kg· (ha^−1^)
MR219	Control	0.76 ± 0.02	2.3 ± 0.16	–	–	–
	Sb16	0.57 ± 0.02	3.6 ± 0.14	0.89 ± 0.16	23	11
	Sb26	0.59 ± 0.01	3.8 ± 0.10	0.84 ± 0.18	22	13

Mayang Segumpal	Control	0.80 ± 0.03	2.2 ± 0.14	–	–	–
	Sb16	0.60 ± 0.01	3.9 ± 0.10	0.93 ± 0.11	22	11
	Sb26	0.58 ± 0.02	4.2 ± 0.10	1.00 ± 0.09	24	12

a.e., atomic excess; Nfda, percent nitrogen derived from atmosphere.

**Table 6:** Diazotrophs isolated from different soil series of Tanjong Karang rice irrigation project area, Malaysia and some of soil chemical properties of isolated soil. Different letters in the columns are significantly different at *p <* 0.05.

SNo.	Soil series/types	Diazotrophs isolated	Soil pH	Organic carbon (%)	Total nitrogen (%)
1	Jawa	Sb1, Sb2, Sb26, Sb28, Sb32	4.71 c	6.67 c	0.44 c
2	Serong	Sb3, Sb4, Sb17, Sb41, Sb42	4.90 b	2.94 e	0.38 d
3	Organic Clay & Muck	Sb43, Sb44, Sb45, Sb46, Sb19, Sb21, Sb47, Sb48, Sb49	4.50 d	9.11 a	0.66 a
4	Sedu	Sb6, Sb16, Sb18, Sb35, Sb37, Sb38	4.40 e	7.89 b	0.55 b
5	Bernam	Sb7, Sb9, Sb12, Sb20, Sb40	4.87 b	5.43 c	0.24 e
6	Bakau	Sb10, Sb13, Sb14, Sb23	5.18 a	3.08 e	0.27 e
7	Brown Clay	Sb15, Sb27, Sb33, Sb34	4.63 d	5.13 d	0.33 d

Means within the same column followed by the same letters are not significantly different at *p* < 0.05.

## Abbreviations

a.e.: atom excess
ANOVA: analysis of variance
ARA: acetylene reduction assay
DGGE: Denaturing Gradient Gel Electrophoresis
PC: principal components
SEM: scanning electron microscopy
TEM: transmission electron microscopy
